# Surgical Site Infections and Antimicrobial Resistance After Cesarean Section Delivery in Rural Rwanda

**DOI:** 10.5334/aogh.3413

**Published:** 2021-08-06

**Authors:** Lotta Velin, Grace Umutesi, Robert Riviello, Moses Muwanguzi, Lisa M. Bebell, Marthe Yankurije, Kara Faktor, Theoneste Nkurunziza, Gilbert Rukundo, Jean de Dieu Gatete, Ivan Emil, Bethany L. Hedt-Gauthier, Fredrick Kateera

**Affiliations:** 1 Program in Global Surgery and Social Change, Harvard Medical School, Boston, MA, USA; 2 Partners In Health, Kigali, Rwanda; 3 Surgery and Public Health, Department of Clinical Sciences Lund, Faculty of Medicine, Lund University, Lund, Sweden; 4 Center for Surgery and Public Health, Brigham and Women’s Hospital, Boston, MA, USA; 5 Massachusetts General Hospital Division of Infectious Diseases, Medical Practice Evaluation Center, and MGH Center for Global Health, Boston, MA, USA; 6 Department of Global Health and Social Medicine, Harvard Medical School, Boston, MA, USA; 7 Epidemiology, Department for Sport and Health Sciences, Technical University of Munich, Munich, Germany; 8 National Reference Laboratory, Kigali, Rwanda

## Abstract

**Background:**

As the volume of surgical cases in low- and middle-income countries (LMICs) increases, surgical-site infections (SSIs) are becoming more prevalent with anecdotal evidence of antimicrobial resistance (AMR), despite a paucity of data on resistance patterns.

**Objectives:**

As a primary objective, this prospective study aimed to describe the epidemiology of SSIs and the associated AMR among women who delivered by cesarean at a rural Rwandan hospital. As secondary objectives, this study also assessed patient demographics, pre- and post-operative antibiotic use, and SSI treatment.

**Methods:**

Women who underwent cesarean deliveries at Kirehe District Hospital between September 23rd, 2019, and March 16th, 2020, were enrolled prospectively. On postoperative day (POD) 11 (+/– 3 days), their wounds were examined. When an SSI was diagnosed, a wound swab was collected and sent to the Rwandan National Reference Laboratory for culturing and antibiotic susceptibility testing.

**Findings:**

Nine hundred thirty women were enrolled, of whom 795 (85.5%) returned for the POD 11 clinic visit. 45 (5.7%) of the 795 were diagnosed with SSI and swabs were collected from 44 of these 45 women. From these 44 swabs, 57 potential pathogens were isolated. The most prevalent bacteria were coagulase-negative staphylococci (n = 12/57, 20.3% of all isolates), and Acinetobacter baumannii complex (n = 9/57, 15.2%). 68.4% (n = 39) of isolates were gram negative; 86.7% if excluding coagulase-negative staphylococci. No gram-negative pathogens isolated were susceptible to ampicillin, and the vast majority demonstrated intermediate susceptibility or resistance to ceftriaxone (92.1%) and cefepime (84.6%).

**Conclusions:**

Bacterial isolates from SSI swab cultures in rural Rwanda predominantly consisted of gram-negative pathogens and were largely resistant to commonly used antibiotics. This raises concerns about the effectiveness of antibiotics currently used for surgical prophylaxis and treatment and may guide the appropriate selection of treatment of SSIs in rural Rwanda and comparable settings.

## Introduction

Cesarean sections (c-sections) are the most common major surgical procedure in the world [1], with 5–15% of all pregnant women needing a c-section to give birth safely [2]. Although the c-section rate in sub-Saharan Africa (SSA) is the lowest in the world, estimated at 7.9% of all deliveries [3], access to surgical care and specifically c-sections is increasing in the region [4].

Surgical site infections (SSIs) are the most common c-section-associated complication, with an estimated 10–48% of women delivering via c-section in SSA developing an SSI [5,6,7,8,9,10]. The World Health Organization (WHO) developed antibiotic prophylactic guidelines for SSI prevention [5], however, adherence to these guidelines appears low in SSA [11] with empirical antibiotic selection often based on drug availability and provider preferences [12].

Antimicrobial resistance (AMR) is also rising in the region [13,14], making SSIs more difficult to treat. Although bacteria naturally evolve and develop resistance to antimicrobial agents, this process is dramatically accelerated by the misuse and overuse of antibiotics [15]. Patients who develop resistant infections are at heightened risk for mortality [16], morbidity, longer hospital stays, and catastrophic health expenditures [14].

Limited research has been done on SSI incidence and risk factors, antibiotic use, and AMR among c-section patients in SSA, with previous studies notably being conducted in urban and/or tertiary settings [6,17,18,19,20,21]. These studies report high levels of AMR and predominantly gram-negative pathogens, suggesting that pathogen and AMR profiles associated with SSIs in low- and middle-income countries (LMICs) may differ from the gram-positive pathogens typically isolated in high-income countries [22]. A study from Rwanda’s main referral hospital similarly demonstrated high prevalence of gram-negative pathogens in wound infections and extensive AMR [23]. This study team aims to describe the epidemiology of SSIs and associated AMR profiles among women delivering by c-section at the Kirehe District Hospital in rural Rwanda. As secondary objectives, this study also assessed patient demographics, pre- and post-operative antibiotic use, and SSI treatment. This study is the first the team is aware of to describe the epidemiology of SSIs and associated AMR profiles among patients at a rural district hospital in Africa, and one of few prospective studies in the region.

## Methods

### Study location

This study was conducted at Kirehe District Hospital located in the Eastern Province of Rwanda, approximately three hours driving distance from the capital city, Kigali. Kirehe District Hospital is a governmental hospital supported by Partners In Health-Rwanda (locally known as Inshuti Mu Buzima) since 2006. The hospital serves a catchment area of 420,000 people including 60,000 residents in the Mahama Refugee Camp. In 2019, Kirehe District Hospital reported 4,560 deliveries, of which 2,715 (59.5%) were c-sections–largely representing the fact that uncomplicated vaginal deliveries take place at health centers rather than in the hospital. The hospital has an obstetrician/gynecologist, and between 11 and 18 general practitioners (GPs), who perform c-sections in the hospital’s one operating theatre.

### Study population, data and sample collection

This study was nested in a larger prospective cohort study following women who delivered via c-section at Kirehe District Hospital. All women who delivered via c-section at Kirehe District Hospital between September 23, 2019, and March 16, 2020 were approached for enrollment, except for December 16–28, when study activities ceased due to the holiday season. Study enrollment was planned until March 22, 2020, but ended eight days early when all non-essential activities were shut down due to the COVID-19 pandemic. All women for whom study-related follow-up was feasible, before COVID-19 distancing policies were implemented, were included. Women from Mahama Refugee Camp were excluded because of the inability to complete study activities due to restricted movement outside the camp.

**Figure 1:**
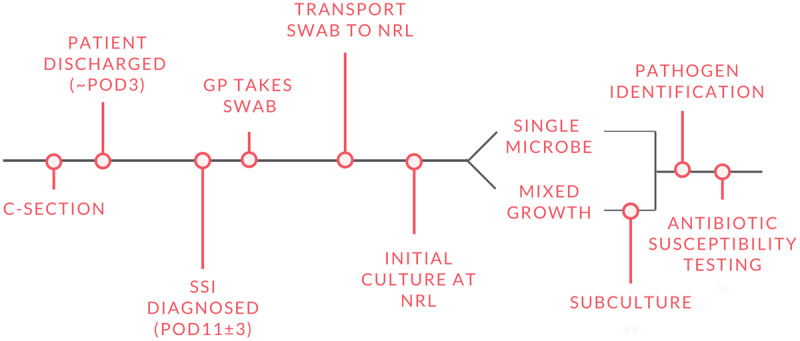
Overview of study processes. C-section:Cesarean section; POD:Post-operative day; SSI:Surgical-site infection; GP:General practitioner; NRL:National Reference Laboratory.

All participants provided written informed consent and were enrolled on the first postoperative day (POD 1). Trained Rwandan study data collectors conducted interviews to collect demographic and clinical data. This data included an Ubudehe category which is a Rwandan socioeconomic classification system where category 1 is the poorest and category 4 the least poor. Additional clinical data were extracted from medical charts. At discharge, an appointment was scheduled for the study clinic (***Figure 1***) for POD 11 (+/– 3 days). This post-operative follow-up window was selected as SSIs most frequently develop between POD 5 and 10 [24], and expected to continue through POD 11.

Study clinics were held twice per week. If a participant missed the first clinic, she was called by telephone and a second visit date was scheduled. If she missed the second visit, she was considered lost to follow-up. Participants received a travel voucher to compensate for the time and the travel costs to attend the study clinic. Women who were not discharged, or who had been readmitted before POD 11, were also scheduled to attend the study clinic.

The study clinic was run by one of two trained GPs, a hospital nurse, and a study member. Before commencing data collection, two study GPs were trained to diagnose SSIs using the Center for Disease Control and Prevention guidelines [25] and to collect swabs. GPs first completed a REDCap survey collecting symptom data related to the surgical wound and then examined the wound for presence of infection. If an SSI was detected, data on infection duration and treatment were collected.

If an SSI was diagnosed, a swab sample was collected using the Levine technique [26]. The GP irrigated the wound using sterile saline and removed excess saline using a sterile gauze pad. A sterile rayon-tipped swab was applied to clean viable tissue at the deepest accessible part of the wound, gently pressed down, and turned to collect expressed fluid. The swab was immediately placed in a tube containing Amies gel transport media, labeled with the patient initials, sample code, and date and time of swab collection.

### Transport and processing of samples

All samples were transported to the Rwanda National Reference Laboratory (NRL) in Kigali within 36 hours of swab collection, using standard transportation procedures (triple packaging and cold chain). The NRL performed initial microbiology cultures, bacterial isolate identification, and antimicrobial susceptibility testing, per NRL standard operating procedures. In high-income settings, agar media made from sheep’s blood is the gold standard for laboratory analysis [27,33]. Due to difficulties procuring sheep’s blood in Rwanda, human blood obtained from the National Transfusion Center was used to prepare blood agar plates, following NRL standard practice.

All cultures and AMR testing were carried out by a laboratory technician trained in the preparation of agar plates, Gram stains, bacterial growth analysis, and safe transportation of biohazard materials. Plates were incubated at 37 degrees Celsius and examined for growth after 24 hours. If no growth was detected, plates were re-incubated for an additional 48 hours before being declared negative. Isolates were identified based on colony morphology, Gram stain, and the VITEK2 testing system. When growth was detected in the initial culture, Gram stain and subcultures were performed using enriched and selective media informed by the characteristics of the organism in the initial culture. Antimicrobial susceptibility for each isolate was determined using the VITEK2 testing system.

### Data analysis and management

All data were stored securely in REDCap. Patient identifiers and confidential information, including name and hospital file number, were kept in a separate password-protected spreadsheet to facilitate reporting of patient results and follow-up per phone in case of missing data.

All data were analyzed using Stata/IC 16.0. First, the overall bacterial profiles for each sample with growth were described. Then, AMR profiles were summarized as the proportion of pathogens which were intermediate or resistant to the tested antibiotic, overall and by Gram category. When available before discharge, patient results were shared with hospital physicians for use in patient care. Participants discharged before getting results were contacted by the study manager in collaboration with hospital health care providers to appropriately adjust treatment plans based on microbiology results.

### Ethics

This study conforms to the principles embodied in the Declaration of Helsinki and was approved by the Rwandan National Ethical Committee (No.326/RNEC/2019) and the Harvard Human Resources Protection Program (IRB18-1033). The study received technical approval from Inshuti Mu Buzima’s Research Committee (IMBRC).

## Results

### Overall patient demographics and antibiotic use

A total of 930 women were enrolled in the study, of whom 85.5% (n = 795) completed the POD 11 study clinic visit. Of the 135 lost-to-follow-up, 66 (48.9%) had their scheduled study clinic visit canceled due to the COVID-19 outbreak. The analysis was restricted to the 795 women for whom study follow-up was completed. The majority (n = 490, 61.6%) were in Ubudehe category 1 or 2. HIV seroprevalence was 0.7%. HIV prevalence and Ubudehe category did not differ significantly by SSI diagnosis.

#### Antibiotic prescriptions

Preoperative antibiotics were given to 88.8% (n = 815) of the participants, most commonly ampicillin (n = 766, 94.0%). Postoperative antibiotics were prescribed to 95.9% (n = 883), most commonly ampicillin (n = 486, 52.3%), followed by gentamicin (n = 447, 48.1%), metronidazole (n = 431, 46.3%), and ceftriaxone (n = 384, 41.3%).

### Details of surgical site infections

Among the 795 women who completed the POD 11 study visit, 45 (5.7%) were diagnosed with an SSI. Most (n = 40, 88.9%) SSIs were described as superficial, and five (11.1%) were deep (***Table 1***). Nearly half of the participants (n = 21, 46.7%) had not noticed the infection prior to diagnosis, 17 (39.5%) had noticed it for 1–3 days and four (9.3%) had noticed it for 4–7 days. Forty-four (97.8%) had open infections that could be swabbed using the Levine technique.

**Table 1:** Characteristics of participants presenting with SSI in study clinic (N = 45), from which 44 had infections that could be swabbed using the Levine Technique.


	n (%)

SSI class	

Superficial	40 (88.9)

Deep	5 (11.1)

Time noticed SSI*	

Never	22 (48.9)

1–3 days	17 (37.8)

4–7 days	4 (8.9)

Other post-operative complication	

Hematoma	3 (6.8)

None	41 (93.2)

SSI Treatment at POD 11 clinic**	

Antibiotics	27 (61.4)

Daily dressings	17 (38.6)

Wound opening	9 (20.5)

Hospital admission	3 (6.8)


* This data was missing from two patients with an SSI. ** Some participants received multiple treatments (N = 14, 31.8%).

#### Pathogens

Of the 44 participants with SSI swabs collected, 77.3% (n = 34) of swab cultures showed single-colony growth while 22.7% (n = 10) had mixed growth of at least two different isolates. In total, 57 potential pathogens were isolated, including 39 (68.4%) gram-negative isolates and 18 (31.6%) gram-positive. The most common gram-negative pathogens (***Table 2***) were *Acinetobacter baumanii* complex (23.1%), *Proteus* spp. (20.5%), *Klebsiella pneumoniae (*20.5%), and *Enterobacter cloacae* complex *(*20.5%). The most prevalent gram-positive bacteria (***Table 3***) were coagulase-negative staphylococci (CoNS, 66.7%), *Staphylococcus aureus* (11.1%), and *Lactococcus lactis* (11.1%).

**Table 2:** Prevalence of gram-negative bacteria in SSI wound isolates and proportion testing intermediate or resistant to each antimicrobial.


GRAM-NEGATIVE, n = 39	CTX n = 38	CEFEP n = 39	AMP n = 39	AMOX/CLAV n = 39	GENT n = 39	AMIK n = 39	CIPRO n = 39	LEVOFLOX* n = 30	PIP n = 39	TETRA n = 39	TMP-SMX n = 39	IMI n = 39

Bacteria												

*E. coli* (n = 3)	100	100	100	0	33	0	33	0	0	66	100	0

*K. pneumoniae* (n = 8)	100	88	100	100	100	0	25	0	75	88	75	0

*Proteus*. spp (n = 8)	88	88	100	25	13	63	63	67	25	100	63	38

*E. cloacae* complex (n = 8)	100	100	–	100	75	13	13	0	50	75	88	13

*A. baumanii complex* (n = 9)	100	78	–	–	56	–	44	0	78	56	67	11

*Pseudomonas*. spp (n = 2)	–	0	–	–	0	0	0	0	0	0	0	0

*S. paucimobilis* n = 1)	100	100	–	–	100	100	100	0	100	100	100	0


CTX:ceftriaxone; Cefep:cefepime; Amp:ampicillin; Amox/clav:amoxicillin-clavulanic acid; Gent:gentamicin; Amik:amikacin; Cipro:ciprofloxacin; Levoflox:levofloxacin; Pip:piperacillin-tazobactam; Tetra:tetracycline; TMP-SMX:trimethoprim-sulfamethoxazole; Imi:Imipenem; *E. Coli:Escherichia coli*; *K. pneumoniae:Klebsiella pneumoniae*; *Proteus spp:Proteus species*; *E. cloacae complex:Enterobacter cloacae complex; A. baumanii complex:Acinetobacter baumanii complex*; *Pseudomonas spp:Pseudomonas species*; *S. Paucimobilis:Sphingomonas paucimobilis*. *Antibiotics that were added to the testing panel 3 months into the study, % resistant/intermediate reported in table is based on number of isolates tested. – indicates “Not Tested”. All (n = 30, 100%) isolates tested were resistant to cefazolin and aztreonam. Of 30 isolates tested, 100% were susceptible to ertapenem, one isolate had intermediate susceptibility towards meropenem. Of 30 isolates tested against nitrofurantoin, 100% of *K. pneumoniae* and *Proteus* spp., 67% of *E. cloacae* complex, and 0% of *E. coli* were intermediate/resistant.

**Table 3:** Prevalence of gram-positive bacteria in SSI wound isolates and proportion testing intermediate or resistant to each antimicrobial.


GRAM-POSITIVE, n = 18	PCN n = 18	OXA* n = 8	CEFOX* n = 8	TMP-SMX n = 18	CLINDA** n = 18	TETRA n = 18	LEVOFLOX* ⤉ n = 8	VANC n = 18	LINEZ* n = 8	RIF* n = 8	TIGE* n = 8	TEICO* n = 8

Bacteria												

*S. aureus* (n = 2)	100	0	100	100	0	50	0	0	0	0	0	0

CoNS (n = 12)	100	71	29	82	55	55	29	0	0	0	0	0

*S. pseudintermedius* (n = 1)	100	–	–	100	100	100	–	100	–	–	–	–

*L. lactis* spp (n = 2)	100	–	–	100	100	100	–	100	–	–	–	–

*K. rhizophilia* (n = 1)	100	–	–	100	100	100	–	100	–	–	–	–


PCN:penicillin; Oxa:oxacillin; Cefox:cefoxitin; TMP-SMX:trimethoprim-sulfamethoxazole; Clinda:clindamycin; Tetra:tetracycline; Levoflox:levofloxacin; Vanc:vancomycin; Linez:linezolid; Rif:rifampicin; Tige:tigercycline; Teico:teicoplanin; *S. aureus:Staphylococcus aureus*; CoNS:coagulase-negative staphylococci; *S. pseudintermedius:Staphylococcus pseudintermedius*: *L. lactis* spp:*Lactococcus lactis* species; *K. rhizophilia:Kocuria rhizophilia*. - indicates “Not Tested.” * Antibiotics that were added to the testing panel 3 months into the study, % resistant/intermediate reported in table is based on number of isolates tested. ** Erythromycin susceptibility was concordant with clindamycin susceptibility except for one CoNS isolate that is clindamycin susceptible but erythromycin resistant. Two CoNS and no *S*. aureus isolates had inducible clindamycin resistance. ⤉ Moxifloxacin susceptibility was fully concordant with levofloxacin susceptibility.

#### Antimicrobial susceptibility

All pathogens demonstrated resistance to at least one antibiotic. Gram-negative pathogens (n = 39) had reduced susceptibility to ampicillin (100% intermediate susceptibility or resistance), ceftriaxone (92.1%), and cefepime (84.6%) (***Table 2***). Both (n = 2, 100%) *S. aureus* isolates were resistant to penicillin and cefoxitin (***Table 3***).

#### SSI treatment

Nearly all (n = 42, 93.3%) participants with an SSI received treatment at the study clinic (***Table 1***). Antibiotic administration was the most common treatment (n = 27, 61.4%), followed by daily dressing changes (n = 17, 38.6%). Twenty-seven (60.0%) patients with an SSI received at least one antibiotic prescription from the study clinic, while six (13.3%) were prescribed two antibiotics. Cloxacillin was the most commonly prescribed antibiotic (n = 17, 63.0%) followed by ceftriaxone (n = 7, 25.9%) and metronidazole (n = 6, 22.2%).

## Discussion

In this prospective study, 5.7% of women delivering by c-section at a rural Rwandan hospital developed an SSI between POD 8–14. Potential pathogens were isolated from all SSI swab cultures, and 77.3% grew a single bacterial isolate. Gram-negative pathogens were the most common, representing 68.4% of all growth, similar to findings in other studies of SSIs and wound infections in SSA [6,17,18,19,20,21,23,28]. The predominantly gram-negative pathogens in this study differ from the historical microbiology of c-section SSI pathogens in high-income settings, where gram-positive bacteria including *S. aureus*, and group A and B streptococcus are more prevalent [22,29].

The majority of gram-positive bacteria isolated in our study were CoNS. Though CoNS can cause infections, especially in immunocompromised patients, they are typically considered skin contaminants [30,31]. If we disregard CoNS, 86.7% of all potential pathogens isolated were gram-negative. The high proportion of gram-negative bacteria could be the result of antibiotic selection pressure from pre- and post-cesarean ampicillin administration, disproportionately killing gram-positive bacteria. Apart from CoNS, the most common pathogens isolated were *A. baumanii, Proteus* spp., *K. pneumoniae*, and *E. cloacae*. These bacteria are often associated with nosocomial infections and high morbidity [32,33], potentially indicating a nosocomial source of SSI. Although not a traditional pathogen in SSIs, recent studies have reported *Acinetobacter* as a cause of SSIs in resource-limited settings [17,34,35,36,37]. Moreover, third generation cephalosporins, including ceftriaxone, are a known risk factor for developing *Acinetobacter* infection, which 4.3% of study participants received pre-operatively and 41.3% postoperatively. All *Acinetobacter, Klebsiella, Proteus*, and *Enterobacter cloacae* isolates in this study demonstrated high levels of AMR, including non-susceptibility to imipenem, an antibiotic not available at Rwandan district hospitals. Another unusual wound isolate was *L. lactis*, only rarely reported as a pathogen in patients consuming unpasteurized milk [38]. To the best of the team’s knowledge, this study is the first to demonstrate *L. lactis* in an SSI swab. This finding could result from the traditional medical practice of using dairy paste on the wound site to promote healing or could also represent contamination and is important to study further.

Similar to other studies in SSA, this study, despite the small sample, indicates likelihood of high rates of AMR [6,17,18,19,20,21]. Although AMR is a global threat, widespread AMR in LMICs like Rwanda is especially concerning as antibiotic availability is more limited than in high-income countries. Although WHO recommends a single dose of pre-cesarean antibiotic prophylaxis to reduce SSI risk [5], we found that they were inconsistently given to study participants. Further, prophylactic postoperative antibiotics, not recommended by WHO, were prescribed in almost all cases. Universal ampicillin resistance among bacterial SSI isolates in this study may be a result of its long-term and frequent use preoperatively, and could indicate the need to reconsider its use as a post-operative prescription also, as it is likely adding to further resistance–with limited clinical benefit. In this study, both *S. aureus* isolates were resistant to cefoxitin, indicating methicillin resistance (MRSA).

This study has several limitations. Firstly, data collection was stopped three weeks early due to the COVID-19 outbreak. As a result, 66 participants were unable to attend the scheduled study clinic on POD 11, leading to a slightly reduced study population. Another limitation is that the postoperative antibiotic use may be inaccurately assessed as adherence to prescriptions was not measured. Furthermore, participants may have also used non-prescribed antibiotics, potentially interfering with microbiology culture results. The usefulness of wound cultures is also debated, as surgical sites are a non-sterile environment, and even when using semi-sterile techniques–as performed in our study–skin colonizers or wound contaminants may be sampled and misinterpreted as pathogens [39]. In this study, several likely non-pathogenic isolates were found, including *Lactococcus, Kocuria*, and CoNS, which could indicate that swabs were contaminated or that the true pathogen or site of infection was not accessed using an appropriate swabbing technique. Although all participants diagnosed clinically with an SSI in our study had growth in microbiology cultures, there is a risk of false-negative culture results due to incorrect swab technique or prior antibiotic treatment [39]. Moreover, because of the need for bacteria to grow in culture and sub-culture before resistance testing can be performed, results may take 36–48 hours to return, limiting their clinical utility at this time [39]. Finally, in this study, five (0.6%) patients received a non-standardized POD 11 exam by a non-study GP due to unavailability of study GPs, and one patient (0.1%) left the clinic before being seen by the doctor. These aberrancies are unlikely to affect our main study findings since they represent <1% of our results.

The extent to which the results in this study can be generalized and applied to SSIs in other LMIC settings is unclear. This study was carried out at a rural district hospital, which serves a largely rural patient population with unique environmental exposures, antibiotic access, and social determinants of health. However, the pathogens isolated, and AMR profiling results are similar to regional and tertiary-level hospitals in the region [17,23,28], suggesting that these findings are likely generalizable to other contexts in the region. Secondly, this team studied SSIs in women who had undergone c-sections, a patient group who, in comparison with the general population, may have a lower HIV seroprevalence, diabetes, and other co-morbidities which would predispose to SSIs or AMR. However, as in other SSA countries, the majority of surgeries performed in Rwanda are c-sections, most of which occur at district hospitals. Thus, these findings likely reflect practices, outcomes, and challenges common across SSA and demonstrate the need for further research on AMR in SSIs and increased antimicrobial stewardship efforts throughout SSA.

## Conclusions

In this first published study on SSI pathogens and AMR profiles of women delivering via c-section in a rural district hospital in Africa, patterns different from those most commonly reported in high-income countries were identified, but were similar to reports from urban and tertiary hospitals in SSA. Pathogens isolated from SSI swab cultures, which were predominantly gram-negative bacteria, differed from those most commonly reported from high-income countries. Findings of high rates of resistance to available antibiotics is consistent with the concerning global trend towards highly resistant pathogens and lack of effective treatment options, especially in a resource-constrained environment. Additional research is needed to determine risk factors contributing to the high prevalence of gram-negative and resistant pathogens identified in order to guide context-appropriate care and mitigate infection risks. These data can inform the development of evidence-based protocols for rational use of antibiotics in SSI prevention and treatment in rural Rwanda and comparable settings.

## Patient and public involvement

Patients and public were not involved in the design of the study. Community health workers were involved in the implementation of the larger study, in which this study was nested, through home visits to patients. Due to COVID-19, dissemination of the study results have not yet involved patients or the public, but the team plans to involve patients and public in dissemination through informing treatment protocols.
